# 1,3-Bis[(3-chloro­pyrazin-2-yl)­oxy]benzene

**DOI:** 10.1107/S160053681301129X

**Published:** 2013-04-30

**Authors:** Thothadri Srinivasan, Venkatesan Kalpana, Perumal Rajakumar, Devadasan Velmurugan

**Affiliations:** a Centre of Advanced Study in Crystallography and Biophysics, University of Madras, Guindy Campus, Chennai 600 025, India; b Department of Organic Chemistry, University of Madras, Guindy Campus, Chennai 600 025, India

## Abstract

The asymmetric unit of the title compound, C_14_H_8_Cl_2_N_4_O_2_, contains one half-mol­ecule, the complete mol­ecule being generated by the operation of a twofold rotation axis. The Cl atom deviates significantly from the plane of the pyrazine ring [0.0215 (4) Å]. The central benzene ring makes a dihedral angle of 72.82 (7)° with the plane of the pyrazine ring.

## Related literature

For applications of the pyrazine ring system in drug development, see: Du *et al.* (2009 ▶); Dubinina *et al.* (2006 ▶); Ellsworth *et al.* (2007 ▶); Mukaiyama *et al.* (2007 ▶). For background to the fluorescence properties of compounds related to the title compound, see: Kawai *et al.* (2001 ▶); Abdullah (2005 ▶). For a related structure, see: Nasir *et al.* (2010 ▶).
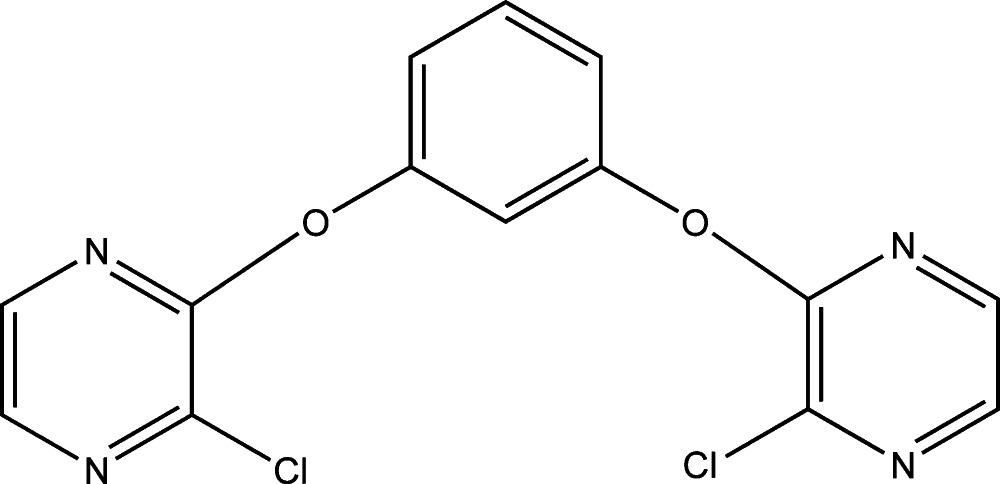


## Experimental

### 

#### Crystal data

C_14_H_8_Cl_2_N_4_O_2_
*M* *_r_* = 335.14
Monoclinic, 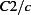

*a* = 9.9618 (3) Å
*b* = 10.2196 (4) Å
*c* = 14.6010 (6) Å
β = 106.231 (2)°
*V* = 1427.22 (9) Å^3^
*Z* = 4
Mo *K*α radiation
μ = 0.47 mm^−1^
*T* = 293 K
0.30 × 0.25 × 0.20 mm

#### Data collection

Bruker SMART APEXII area-detector diffractometer
Absorption correction: multi-scan (*SADABS*; Bruker, 2008 ▶) *T*_min_ = 0.873, *T*_max_ = 0.912
6736 measured reflections
1781 independent reflections
1552 reflections with *I* > 2σ(*I*)
*R*_int_ = 0.026

#### Refinement

*R*[*F* ^2^ > 2σ(*F* ^2^)] = 0.036
*wR*(*F* ^2^) = 0.113
*S* = 1.00
1781 reflections
101 parameters
H-atom parameters constrained
Δρ_max_ = 0.25 e Å^−3^
Δρ_min_ = −0.31 e Å^−3^

### 

Data collection: *APEX2* (Bruker, 2008 ▶); cell refinement: *SAINT* (Bruker, 2008 ▶); data reduction: *SAINT*; program(s) used to solve structure: *SHELXS97* (Sheldrick, 2008 ▶); program(s) used to refine structure: *SHELXL97* (Sheldrick, 2008 ▶); molecular graphics: *ORTEP-3 for Windows* (Farrugia, 2012 ▶) and *Mercury* (Macrae *et al.*, 2008 ▶); software used to prepare material for publication: *SHELXL97*and *PLATON* (Spek, 2009 ▶).

## Supplementary Material

Crystal structure: contains datablock(s) global, I. DOI: 10.1107/S160053681301129X/kp2450sup1.cif

Structure factors: contains datablock(s) I. DOI: 10.1107/S160053681301129X/kp2450Isup2.hkl

Supplementary material file. DOI: 10.1107/S160053681301129X/kp2450Isup3.cml

Additional supplementary materials:  crystallographic information; 3D view; checkCIF report

## Figures and Tables

**Fig. 1.**
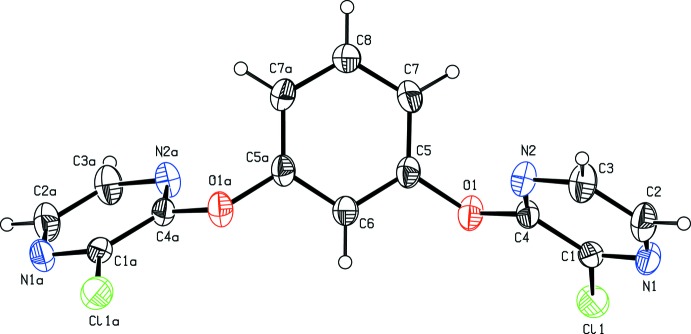
The molecular structure of the title compound, showing displacement ellipsoids drawn at the 30% probability level. H atoms are presented as small spheres of arbitrary radius. The related atoms have the symmetry code: (a) -x, y, -z+1/2.

## supplementary crystallographic information

### Comment

The pyrazine ring system is a useful structural element in medicinal chemistry and has found broad applications in drug development which can be used as antiproliferative agent (Dubinina *et al.*, 2006), potent CXCR3 antagonists (Du *et al.*, 2009), CB1 antagonists (Ellsworth *et al.*, 2007), and c-Src inhibitory (Mukaiyama *et al.*, 2007). On-going structural studies of heterocyclic N-containing derivatives (Nasir *et al.*, 2010) are motivated by an investigation of their fluorescence properties (Kawai *et al.*, 2001; Abdullah, 2005). In view of different applications of this class of compounds, we have undertaken the single crystal structure determination of the title compound.

The title compound C_14_ H_8_ Cl_2_ N_4_ O_2_, contains a half of the molecule in an asymmetric unit; the complete molecule is generated by two the fold rotation axis along the direction [0 1 0] with the symmetry code: -x, y, -z+1/2. X-ray analysis confirms the molecular structure and atom connectivity of the compound (Fig. 1). The deviation of the atom Cl1 from the pyrazine ring (C1/N1/C2/C3/N2/C4) is -0.0215 (4) Å.

The central phenyl ring (C5/C6/C7/C8/C5^i^/C7^i^) forms the dihedral angle of 72.82 (7) ° with the pyrazine ring (C1/N1/C2/C3/N2/C4). The dihedral angle between the pyrazine rings (C1/N1/C2/C3/N2/C4) and (C1^i^/N1^i^/C2^i^/C3^i^/N2^i^/C4^i^) is 68.38 (3) ° (Macrae *et al.*, 2008). The crystal packing is via van der Waals interactions, only.

### Experimental

To a stirred solution of Cs_2_CO_3_/K_2_CO_3_ (22 mmol) in CH_3_CN (50 mL), resorcinol (10 mmol) was added and stirred for 5 min. 2,3-dichloropyrazine (20 mmol) in CH_3_CN (100 mL) was added dropwise to the above reaction mixture and allowed for stirring at refluxing condition for 12 h. After the reaction was complete, the reaction mixture was allowed to attain room temperature and then evaporated to dryness. The residue obtained was extracted with CH_2_Cl_2_ (3 x 100 mL), washed with water (3 x 100 mL), brine and then dried over Na_2_SO_4_. Evaporation of the organic layer gave a residue, which on purification using column chromatography with hexane/CHCl_3_ (1:1) as an eluent gave the corresponding compound. Single crystals suitable for X-ray diffraction were obtained by slow evaporation of a solution of the title compound in hexane at room temperature.

### Refinement

The hydrogen atoms were placed in calculated positions with C—H = 0.93 Å, refined in the riding model with fixed isotropic displacement parameters:*U*_iso_(H) = 1.2*U*_eq_(C).

### Crystal data

**Table:**

C_14_H_8_Cl_2_N_4_O_2_	*F*(000) = 680
*M**_r_* = 335.14	*D*_x_ = 1.560 Mg m^−^^3^
Monoclinic, *C*2/*c*	Mo *K*α radiation, λ = 0.71073 Å
Hall symbol: -C 2yc	Cell parameters from 1781 reflections
*a* = 9.9618 (3) Å	θ = 2.9–28.4°
*b* = 10.2196 (4) Å	µ = 0.47 mm^−^^1^
*c* = 14.6010 (6) Å	*T* = 293 K
β = 106.231 (2)°	Block, colourless
*V* = 1427.22 (9) Å^3^	0.30 × 0.25 × 0.20 mm
*Z* = 4	

### Data collection

**Table:**

Bruker SMART APEXII area-detector diffractometer	1781 independent reflections
Radiation source: fine-focus sealed tube	1552 reflections with *I* > 2σ(*I*)
Graphite monochromator	*R*_int_ = 0.026
ω and φ scans	θ_max_ = 28.4°, θ_min_ = 2.9°
Absorption correction: multi-scan (*SADABS*; Bruker, 2008)	*h* = −13→13
*T*_min_ = 0.873, *T*_max_ = 0.912	*k* = −13→12
6736 measured reflections	*l* = −19→14

### Refinement

**Table:**

Refinement on *F*^2^	Primary atom site location: structure-invariant direct methods
Least-squares matrix: full	Secondary atom site location: difference Fourier map
*R*[*F*^2^ > 2σ(*F*^2^)] = 0.036	Hydrogen site location: inferred from neighbouring sites
*wR*(*F*^2^) = 0.113	H-atom parameters constrained
*S* = 1.00	*w* = 1/[σ^2^(*F*_o_^2^) + (0.0685*P*)^2^ + 0.5533*P*] where *P* = (*F*_o_^2^ + 2*F*_c_^2^)/3
1781 reflections	(Δ/σ)_max_ < 0.001
101 parameters	Δρ_max_ = 0.25 e Å^−^^3^
0 restraints	Δρ_min_ = −0.31 e Å^−^^3^

### Special details

**Table:**

Geometry. All esds (except the esd in the dihedral angle between two l.s. planes) are estimated using the full covariance matrix. The cell esds are taken into account individually in the estimation of esds in distances, angles and torsion angles; correlations between esds in cell parameters are only used when they are defined by crystal symmetry. An approximate (isotropic) treatment of cell esds is used for estimating esds involving l.s. planes.
Refinement. Refinement of F^2^ against ALL reflections. The weighted R-factor wR and goodness of fit S are based on F^2^, conventional R-factors R are based on F, with F set to zero for negative F^2^. The threshold expression of F^2^ > 2sigma(F^2^) is used only for calculating R-factors(gt) etc. and is not relevant to the choice of reflections for refinement. R-factors based on F^2^ are statistically about twice as large as those based on F, and R- factors based on ALL data will be even larger.

### Fractional atomic coordinates and isotropic or equivalent isotropic displacement parameters (Å^2^)

**Table:**

	*x*	*y*	*z*	*U*_iso_*/*U*_eq_	
C1	0.10770 (15)	0.37514 (13)	−0.02670 (10)	0.0461 (3)	
C2	−0.07466 (18)	0.3213 (2)	−0.15100 (11)	0.0644 (4)	
H2	−0.1203	0.3246	−0.2159	0.077*	
C3	−0.13428 (17)	0.25363 (19)	−0.09216 (11)	0.0618 (4)	
H3	−0.2196	0.2121	−0.1178	0.074*	
C4	0.04654 (14)	0.30517 (13)	0.03426 (9)	0.0417 (3)	
C5	0.05477 (14)	0.23121 (15)	0.18814 (9)	0.0454 (3)	
C6	0.0000	0.3013 (2)	0.2500	0.0436 (4)	
H6	0.0000	0.3923	0.2500	0.052*	
C7	0.05777 (19)	0.09686 (17)	0.18801 (10)	0.0597 (4)	
H7	0.0979	0.0516	0.1470	0.072*	
C8	0.0000	0.0305 (2)	0.2500	0.0694 (7)	
H8	0.0000	−0.0605	0.2500	0.083*	
N1	0.04795 (16)	0.38337 (13)	−0.11809 (9)	0.0580 (3)	
N2	−0.07257 (13)	0.24545 (13)	0.00221 (8)	0.0515 (3)	
O1	0.11655 (11)	0.30189 (12)	0.12853 (7)	0.0537 (3)	
Cl1	0.26460 (5)	0.45456 (4)	0.01959 (4)	0.06883 (19)	

### Atomic displacement parameters (Å^2^)

**Table:**

	*U*^11^	*U*^22^	*U*^33^	*U*^12^	*U*^13^	*U*^23^
C1	0.0581 (7)	0.0383 (6)	0.0511 (8)	0.0030 (5)	0.0308 (6)	−0.0006 (5)
C2	0.0681 (10)	0.0875 (12)	0.0390 (7)	0.0107 (9)	0.0175 (7)	0.0107 (7)
C3	0.0527 (8)	0.0885 (12)	0.0432 (8)	−0.0002 (8)	0.0121 (6)	0.0067 (7)
C4	0.0491 (6)	0.0447 (7)	0.0363 (6)	0.0042 (5)	0.0201 (5)	0.0004 (5)
C5	0.0493 (7)	0.0565 (8)	0.0298 (6)	−0.0035 (5)	0.0104 (5)	0.0000 (5)
C6	0.0461 (9)	0.0492 (10)	0.0340 (8)	0.000	0.0085 (7)	0.000
C7	0.0861 (11)	0.0587 (9)	0.0385 (7)	0.0055 (8)	0.0240 (7)	−0.0053 (6)
C8	0.119 (2)	0.0472 (12)	0.0468 (12)	0.000	0.0308 (13)	0.000
N1	0.0756 (8)	0.0596 (8)	0.0484 (7)	0.0084 (6)	0.0332 (6)	0.0117 (6)
N2	0.0489 (6)	0.0689 (8)	0.0387 (6)	−0.0036 (5)	0.0157 (5)	0.0063 (5)
O1	0.0553 (6)	0.0703 (7)	0.0374 (5)	−0.0130 (5)	0.0162 (4)	−0.0022 (4)
Cl1	0.0778 (3)	0.0608 (3)	0.0815 (4)	−0.02286 (19)	0.0447 (2)	−0.01437 (19)

### Geometric parameters (Å, º)

**Table:**

C1—N1	1.303 (2)	C5—C7	1.373 (2)
C1—C4	1.4064 (18)	C5—C6	1.3789 (17)
C1—Cl1	1.7229 (15)	C5—O1	1.3991 (17)
C2—N1	1.340 (2)	C6—C5^i^	1.3789 (17)
C2—C3	1.362 (2)	C6—H6	0.9300
C2—H2	0.9300	C7—C8	1.379 (2)
C3—N2	1.345 (2)	C7—H7	0.9300
C3—H3	0.9300	C8—C7^i^	1.379 (2)
C4—N2	1.2996 (18)	C8—H8	0.9300
C4—O1	1.3584 (16)		
			
N1—C1—C4	121.76 (14)	C6—C5—O1	117.57 (14)
N1—C1—Cl1	118.42 (11)	C5—C6—C5^i^	117.45 (19)
C4—C1—Cl1	119.81 (11)	C5—C6—H6	121.3
N1—C2—C3	121.90 (14)	C5^i^—C6—H6	121.3
N1—C2—H2	119.0	C5—C7—C8	118.51 (15)
C3—C2—H2	119.0	C5—C7—H7	120.7
N2—C3—C2	121.51 (15)	C8—C7—H7	120.7
N2—C3—H3	119.2	C7^i^—C8—C7	121.1 (2)
C2—C3—H3	119.2	C7^i^—C8—H8	119.4
N2—C4—O1	120.78 (11)	C7—C8—H8	119.4
N2—C4—C1	121.62 (12)	C1—N1—C2	116.50 (13)
O1—C4—C1	117.60 (12)	C4—N2—C3	116.71 (13)
C7—C5—C6	122.18 (14)	C4—O1—C5	116.90 (10)
C7—C5—O1	120.12 (13)		
			
N1—C2—C3—N2	−0.1 (3)	C4—C1—N1—C2	−0.2 (2)
N1—C1—C4—N2	0.0 (2)	Cl1—C1—N1—C2	−179.30 (12)
Cl1—C1—C4—N2	179.08 (11)	C3—C2—N1—C1	0.3 (3)
N1—C1—C4—O1	179.94 (13)	O1—C4—N2—C3	−179.81 (14)
Cl1—C1—C4—O1	−0.95 (17)	C1—C4—N2—C3	0.2 (2)
C7—C5—C6—C5^i^	−1.04 (11)	C2—C3—N2—C4	−0.1 (3)
O1—C5—C6—C5^i^	−176.94 (13)	N2—C4—O1—C5	0.4 (2)
C6—C5—C7—C8	2.0 (2)	C1—C4—O1—C5	−179.61 (12)
O1—C5—C7—C8	177.84 (11)	C7—C5—O1—C4	75.09 (18)
C5—C7—C8—C7^i^	−0.99 (11)	C6—C5—O1—C4	−108.92 (13)

Symmetry code: (i) −*x*, *y*, −*z*+1/2.

## References

[bb1] Abdullah, Z. (2005). *Intl J. Chem. Sci.* **3** , 9–15.

[bb2] Bruker (2008). *APEX2*, *SAINT* and *SADABS* Bruker AXS Inc., Madison, Wisconsin, USA.

[bb3] Du, X. H., Gustin, D. J., Chen, X. Q., Duquette, J., McGee, L. R., Wang, Z. L., Ebsworth, K., Henne, K., Lemon, B., Ma, J., Miao, S. C., Sabalan, E., Sullivan, T. J., Tonn, G., Collins, T. L. & Medina, J. C. (2009). *Bioorg. Med. Chem. Lett.* **19**, 5200–5204. 10.1016/j.bmcl.2009.07.021 19631529

[bb4] Dubinina, G. G., Platonov, M. O., Golovach, S. M., Borysko, P. O., Tolmachov, A. O. & Volovenko, Y. M. (2006). *Eur. J. Med. Chem.* **41**, 727–737. 10.1016/j.ejmech.2006.03.019 16675067

[bb5] Ellsworth, B. A., Wang, Y., Zhu, Y. H., Pendri, A., Gerritz, S. W., Sun, C. Q., Carlson, K. E., Kang, L. Y., Baska, R. A., Yang, Y. F., Huang, Q., Burford, N. T., Cullen, M. J., Johnghar, S., Behnia, K., Pelleymounter, M. A., Washburn, W. N. & Ewing, W. R. (2007). *Bioorg. Med. Chem. Lett.* **17**, 3978–3982. 10.1016/j.bmcl.2007.04.087 17513109

[bb6] Farrugia, L. J. (2012). *J. Appl. Cryst.* **45**, 849–854.

[bb7] Kawai, M., Lee, M. J., Evans, K. O. & Norlund, T. (2001). *J. Fluoresc* **11**, 23–32.

[bb8] Macrae, C. F., Bruno, I. J., Chisholm, J. A., Edgington, P. R., McCabe, P., Pidcock, E., Rodriguez-Monge, L., Taylor, R., van de Streek, J. & Wood, P. A. (2008). *J. Appl. Cryst.* **41**, 466–470.

[bb9] Mukaiyama, H., Nishimura, T., Kobayashi, S., Ozawa, T., Kamada, N., Komatsu, Y., Kikuchi, S., Oonota, H. & Kusama, H. (2007). *Bioorg. Med. Chem. Lett.* **15**, 868–885. 10.1016/j.bmc.2006.10.041 17095233

[bb10] Nasir, S. B., Abdullah, Z., Fairuz, Z. A., Ng, S. W. & Tiekink, E. R. T. (2010). *Acta Cryst.* E**66**, o2187. 10.1107/S160053681003014X PMC3007539 21588464

[bb11] Sheldrick, G. M. (2008). *Acta Cryst.* A**64**, 112–122. 10.1107/S0108767307043930 18156677

[bb12] Spek, A. L. (2009). *Acta Cryst.* D**65**, 148–155. 10.1107/S090744490804362X PMC2631630 19171970

